# Timelines of infection and transmission dynamics of H1N1pdm09 in swine

**DOI:** 10.1371/journal.ppat.1008628

**Published:** 2020-07-24

**Authors:** Laetitia Canini, Barbara Holzer, Sophie Morgan, Johanneke Dinie Hemmink, Becky Clark, sLoLa Dynamics Consortium, Mark E. J. Woolhouse, Elma Tchilian, Bryan Charleston

**Affiliations:** 1 Usher Institute, The University of Edinburgh, Edinburgh, United Kingdom; 2 Mucosal immunology, Pirbright Institute, Woking, United Kingdom; 3 Viral immunology, Pirbright Institute, Woking, United Kingdom; AMC, University of Amsterdam, NETHERLANDS

## Abstract

Influenza is a major cause of mortality and morbidity worldwide. Despite numerous studies of the pathogenesis of influenza in humans and animal models the dynamics of infection and transmission in individual hosts remain poorly characterized. In this study, we experimentally modelled transmission using the H1N1pdm09 influenza A virus in pigs, which are considered a good model for influenza infection in humans. Using an experimental design that allowed us to observe individual transmission events occurring within an 18-hr period, we quantified the relationships between infectiousness, shed virus titre and antibody titre. Transmission event was observed on 60% of occasions when virus was detected in donor pig nasal swabs and transmission was more likely when donor pigs shed more virus. This led to the true infectious period (mean 3.9 days) being slightly shorter than that predicted by detection of virus (mean 4.5 days). The generation time of infection (which determines the rate of epidemic spread) was estimated for the first time in pigs at a mean of 4.6 days. We also found that the latent period of the contact pig was longer when they had been exposed to smaller amount of shed virus. Our study provides quantitative information on the time lines of infection and the dynamics of transmission that are key parts of the evidence base needed to understand the spread of influenza viruses though animal populations and, potentially, in humans.

## Author summary

Influenza is a major cause of mortality and morbidity worldwide. The relationship between the time course of influenza infection and virus shedding and onward transmission of the virus remains poorly characterized. Pigs are a natural host for influenza infection with shedding patterns similar to humans. Therefore we experimentally infected pigs with the H1N1pdm09 influenza A virus using direct contact challenge and then mixed the infected pigs with a different naïve pig each day to understand when transmission occurred. Using mathematical modeling, we found that transmission events occurred on 60% of occasions when the infected pigs were shedding virus and that the risk of transmission increased with the quantity of virus shed. Also it was clear the incontact pigs started to shed virus later after exposure when the infected pigs were shedding low quantities of virus. Our study therefore provides quantitative information on the time lines of influenza virus infection and the dynamics of transmission. This is important to understand the spread of influenza viruses through animal populations and, potentially, in humans.

## Introduction

The 2009 H1N1 pandemic demonstrated the potential of swine origin influenza viruses to cause significant human morbidity and mortality globally [1–3]. Swine influenza viruses (SwIV) are also of great economic importance for the pig industry as they contribute to the respiratory disease complex and can cause severe pulmonary distress, growth retardation and sub-optimal reproductive performance [4–6]. SwIV are distributed worldwide and are endemic in US and European herds, putting pig industry workers at increased risk of infection [1,2,7]. The pandemic A/H1N1 2009 strain (H1N1pdm09) represented 10% of SwIV isolations in Europe from 2010–2013 [8], while in the UK 42% of pigs tested were seropositive to H1N1 pdm09 [3].

Understanding SwIV transmission is pivotal to managing the risk of spread between pigs and spillover to humans. However, despite the common occurrence of H1N1pdm09 in pigs there is limited information on the viral kinetics and parameters determining influenza A virus transmission between pigs. Transmission models used to predict epidemics rely on natural history parameters that describe the time-course of influenza, such as the latent period or the duration of infectiousness. In addition, it has been previously assumed that infectiousness (which is the ability of an infected individual to transmit a pathogen) is proportional to viral titre. However, this hypothesis has never been tested to our knowledge. It is therefore crucial to provide estimates of natural history parameters and to investigate the link between shed virus titre and the probability of transmission.

Because the pig is an important natural host for influenza A viruses and a source of new pandemic viruses, it is also an appropriate large animal model for human influenza. Pigs and humans are infected by the same subtypes of virus and have similar distributions of sialic acid receptors in the respiratory tract [9,10]. The pig is genetically, immunologically and physiologically more similar to humans than small animals [11]. Furthermore, infection dynamics are similar between the two species. Experimental influenza virus infection lasts on average 1.3 to 7.0 days in humans depending on the infectiousness threshold [12] and 2.0 to 7.5 days in influenza virus naive pigs depending on route of challenge [13]. Pigs are therefore a suitable model to study influenza virus infection and transmission [14,15]. We have previously shown that SwIV viral shedding, infectivity, immune response and pathology depends on the inoculated dose and route of infection [13]. It is therefore important to design studies mimicking natural infection, i.e. infection by contact, to develop a quantitative understanding of SwIV infection dynamics and transmission.

Here, we used an experimental design in which experimental SwIV transmission was analyzed in pigs infected by contact. In our study, a series of individual recipient pigs were placed in contact for a short time with a donor pig also infected by contact. This allows us to follow the time course of a contact acquired influenza virus infection including, precise information on when an infected donor is infectious to a susceptible recipient. We analyzed data on viral and antibody titres to identify determinants of transmission between pigs and to quantify the infection kinetics of naturally acquired H1N1pdm09 influenza in pigs.

## Results

### Transmission experiments

**Fig 1:**
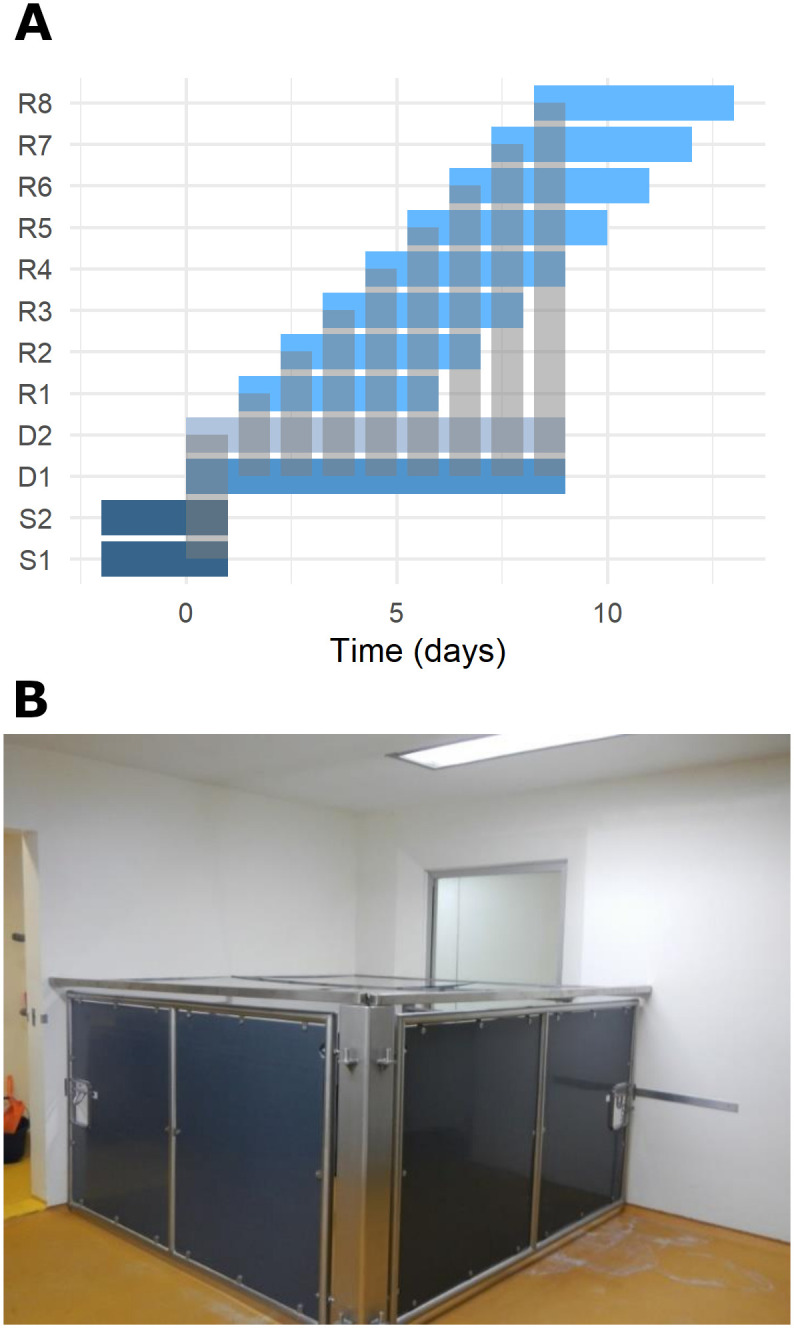
Experimental design. A) Two seeder (S1 and S2) pigs were infected with MAD device and put in contact 2 days later with two donor (D1 and D2) pigs. After 24 h the seeders pigs were culled and one of the donor pig (D1) is put in contact with a different recipient pig (R1-R8) for 18hr (between 2 pm and 8 am). During the remaining 6hr, D1 is co-housed with donor pig 2 (D2). Recipient pigs were sampled daily during 6 days. Each grey rectangle shows a contact, between S and D and between D and R. B) Contact chamber in which donor and recipients were co-housed for 18 hours every day.

**Fig 2:**
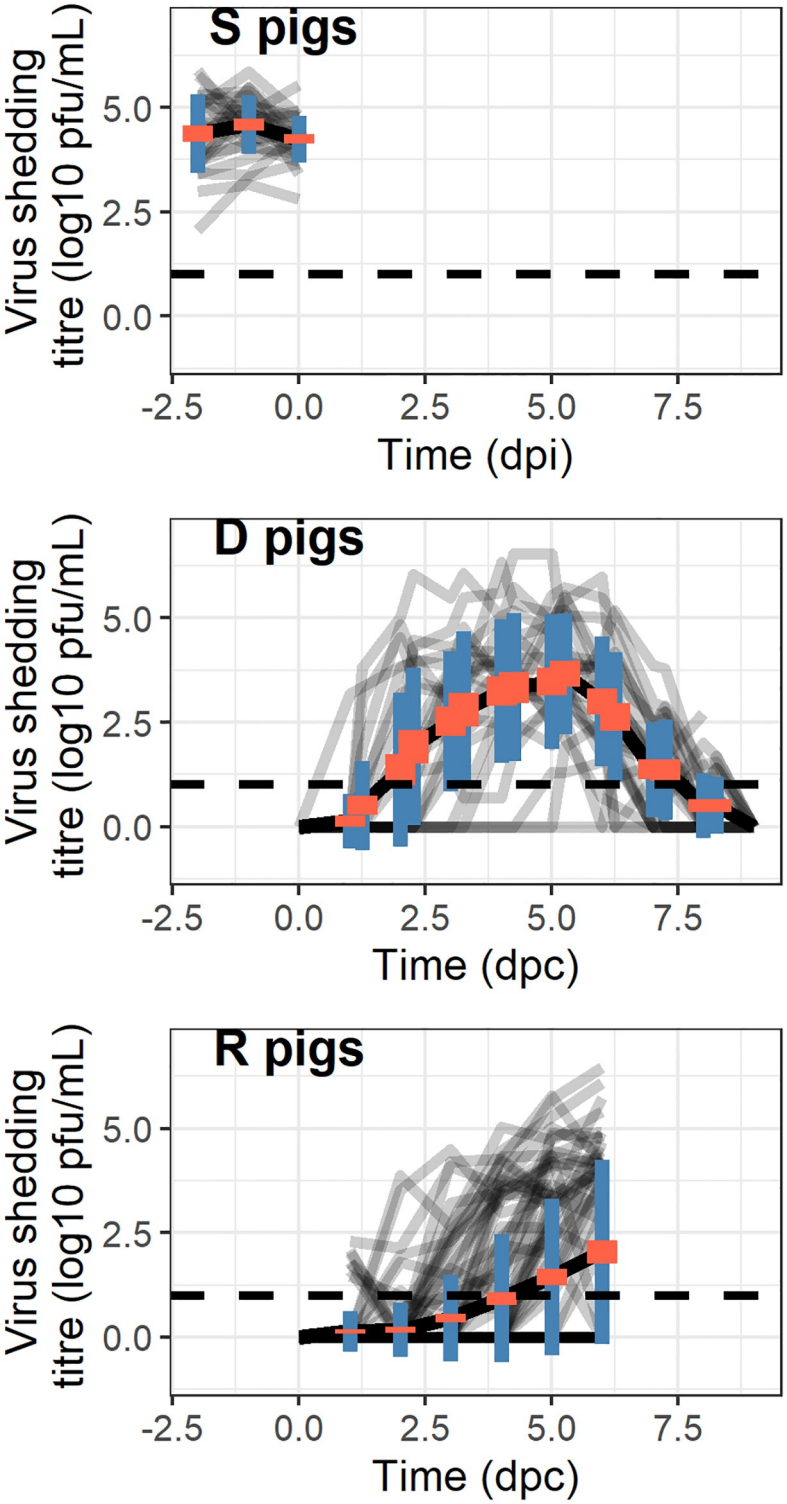
Individual viral kinetics for seeder pigs (upper left panel), donor pigs (lower left panel) and recipient pigs (right panel). Each grey line represents an individual pig. The black line represents the mean viral shedding titre at each sample time. The error bars represent the standard error of the mean. For the seeder pigs time represents the day post inoculation (dpi) whereas for the donors and recipients time represents day post contact (dpc) with the seeder and donor, respectively. For the recipient pigs, the color of the lines represents the day of contact between the donor and the recipient (since the infection of the donor). The dashed line represents the limit of detection.

Twelve individual transmission experiments were performed. During each experiment, two “seeder” (S) pigs were intranasally inoculated with A/swine/England/1353/2009 virus (H1N1pmd09). Two days post inoculation (dpi), the two S pigs were put in contact with two “donor” (D) pigs for 24 hr to allow natural infection. After the 24 h exposure, one D pig was chosen randomly, designated as donor 1 (D1), and placed in contact with a different naïve recipient (R) pig each day for 18 hours during an 8 day period (recipients R1 to R8) (Fig 1). The second D pig, designated as D2, was a companion pig for D1 on welfare grounds. In total, we collected samples from 133 pigs: 24 S pigs, 21 D pigs and 88 R pigs. Three D pigs (1 in contact with R pigs and 2 companion donors) and 8 R pigs (in contact with the excluded D pig) were excluded from the analysis as they presented with detectable levels of antibody at baseline (S1 Table). Full viral kinetics and antibody increase patterns were observed only in D pigs. Only partial viral kinetics are available for S and R pigs as they were euthanized before the end of shedding (Fig 2).

**Fig 3:**
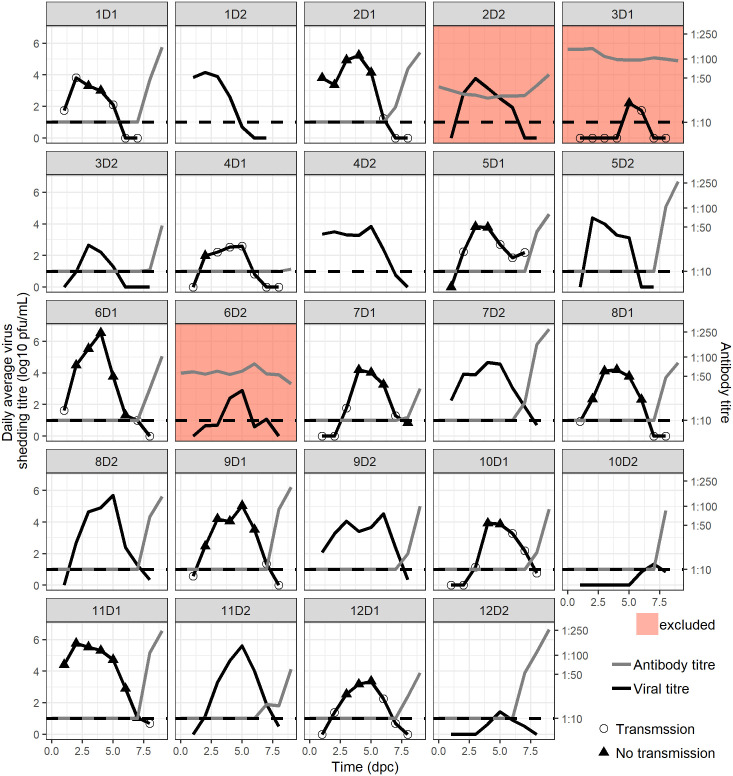
Individual donor and recipient pigs’ infection kinetics. Each box represents a donor pig. The viral kinetics is represented by a black line. Black triangles represents the average daily shed virus titres on days with transmission event and open circles represent the average daily shed virus titres on day without transmission events. The time since separation of seeder and donor is shown on the x axis. Antibody titers are represented by the grey line. The red filled boxes represent the pigs with baseline antibodies that were excluded from the analysis.

We recorded a transmission event when a R pig shed virus above the limit of detection at least once. All donor pigs transmitted influenza virus at least once during the 8 days of follow-up (Fig 3 & S2 Fig). The number of transmission events ranged between 1 (transmission experiment 4) and 7 (transmission experiment 11). The average number of transmission events per donor pig was 3.7 ± 0.5 (Table 1 & S1 Fig). In total, 24/24 S pigs, 21/21 D pigs and 43/88 R pigs were infected.

**Table 1:** Viral kinetics and transmission events description and predicted from viral kinetic model coupled to the Bayesian logistic regression prediction. For quantitative variables: mean ±se [range], for qualitative variables: number or percentage. Dpi stands for days post inoculation and dpc for days post contact. Predictions: 100 replicates of 11 pigs datasets: average of replicates mean, {5^th^-95^th^ percentiles of replicates mean}—Not observed; * estimated for 21 donors; ** estimated for the 11 donors in contact with recipients; £: estimated for 41 infected recipients.† fixed in the model.

	Observed			Predicted (100 replicates)	Relative Bias (%)
Pig type	Seeders	Donors	Recipients	Donors	
Number of pigs	24	21	88	11†	
Exposed animals who become infected–%	100	100	47	85.8 {72.7–100}	-14.2
Latent period (dpi/dpc)	<1	2.54±0.28 [1.00–6.25]	3.22 [1.00–6.00]^£^	2.20 {1.49–2.70}	-15.4
* Time to last detected virus titre (dpc)	-	7.05±0.28 [5.00–8.25]	-	7.52 {6.56–8.76}	+6.7
*Duration of shedding (days)	-	4.51±0.31 [1.25–7.00]	-	5.33 {4.19–6.88}	+18.2
*Time to maximal shed virus titre: Tmax (dpc)	-	4.51±0.32[2.00–8.00]	-	4.43 {3.81–5.20}	-1.8
*Maximal shed virus titre: Vmax (log_10_ pfu/mL)	-	4.58±0.29 [1.81–6.54]	-	5.49 {4.27–6.97}	+19.9
* Generation time: Tg (days)	-	4.62±0.26 [2.73–7.42]	-	4.17 {3.56–4.75}	-9.7
**Time to 1st transmission event (dpc)	-	2.27±0.33 [1.00–4.00]	-	1.85{0–5.23}	-18.5
**Transmission period (days)	-	3.91±0.49 [1.00–6.00]	-	3.54 {0–6.00}	-9.5
**Number of transmission events	-	3.73±0.49 [1.00–6.00]	-	4.13{1.00–9.30}	+10.7
*Onset of antibody production (dpc)	-	7.84±0.12 [7.00–9.00]	-	7.55 {7.18–8.09}	-3.7

### Virus shedding and antibody response

**Fig 4:**
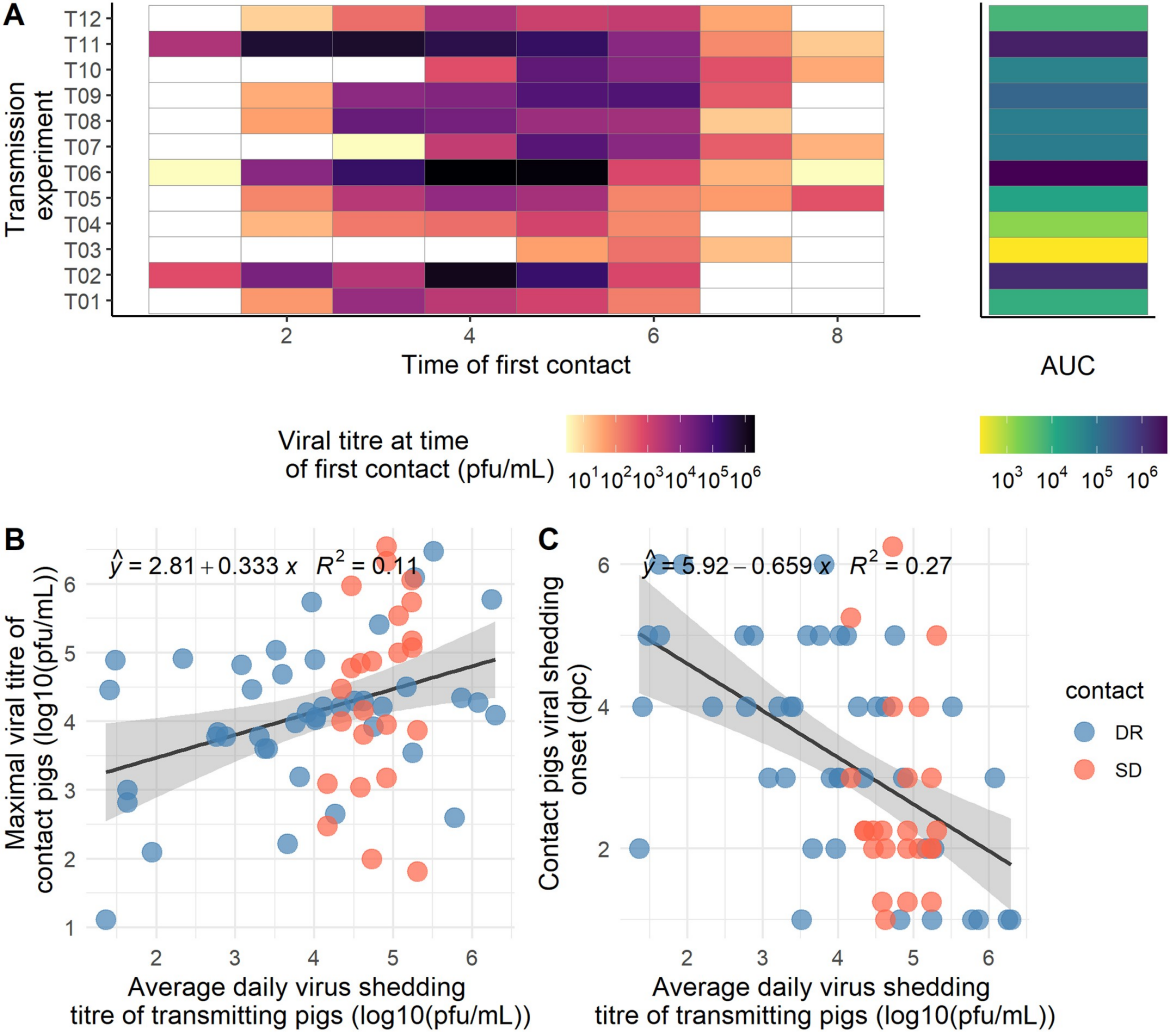
Transmission events and contact shedding. A. Each row represents a transmission experiment. The color of the tiles from the main panel represent the viral titre of the contact donor pig at the start of the in contact period. The side panel represent the corresponding area under the curve (AUC) in log scale. Transmission experiment T03 was excluded due to the presence of antibody in the contact donor pigs at the start of the experiment. B. Contact pig maximal viral titre vs. average daily virus shedding of parent pigs. C. Contact pig viral shedding onset vs. average daily virus shedding of parent pigs. SD and DR stand for Seeder-Donor and Donor-Recipient contact, respectively.

The average daily shed virus titre was above the limit of detection in D pigs for 67/88 D-R contacts. Among these 67 contacts where the D pig was shedding virus, transmission to R pigs occurred on 42/67 (60%) occasions. Only one R pig was infected after being in contact with D pigs with undetected virus shedding: 5R1 (before the onset of detected shedding) (Table 2, Figs 3 and 4A). The number of transmission events correlates with the area under the curve (AUC) of the donor shed virus titre (Kendall’s ρ = 0.94, P<0.0001) (Fig 4A and S2 Fig). On average the first transmission event was observed at 2.27 ± 0.33 dpc (range: 1–4), which is similar to the latent period (2.54±0.30 dpc), which was defined as the time interval between time of inoculation for S pigs or time of first contact for D and R pigs and the time when virus titre was first detected (Table 1).

**Table 2:** Bayesian logistic regression result. 95% CI stands for 95% credibility interval. $\hat{R}$ is a convergence diagnostic that compares the between- and within-chain estimates for model parameters. We considered that model converged if all $\hat{R}$ are <1.01. ESS stands for effective sample size. We considered that model converged if all ESS > 400.

	Odds [95% CI]	$\hat{R}$	ESS
Standardized average shed virus titre during the contact period for contact donor (D1) pig—in log scale	57.0 [10.6–377]	1.00	25840
Standardized contact donor (D1) pig antibody titre at the beginning of contact period	0.171 [0.002–1.92]	1.00	13893
Standardized recipient pig age at the beginning of the contact period	0.259 [0.072–0.899]	1.00	23315
Standardized breed (of D and R pigs)	0.555 [0.138–1.92]	1.00	14769

When virus is detected, transmission events occur more frequently when antibody titre is ≤1:10 than when antibody titre is >1:10 at the first time of contact with 39 out of 56 (69.6%) vs. 1 out of 11 (9.1%) transmission events respectively. When virus is not detected, only one out of 10 (10%) transmission event was observed when antibody titre ≤1:10 and none when antibody titre > 1:10.

### Prediction of transmission events

**Fig 5:**
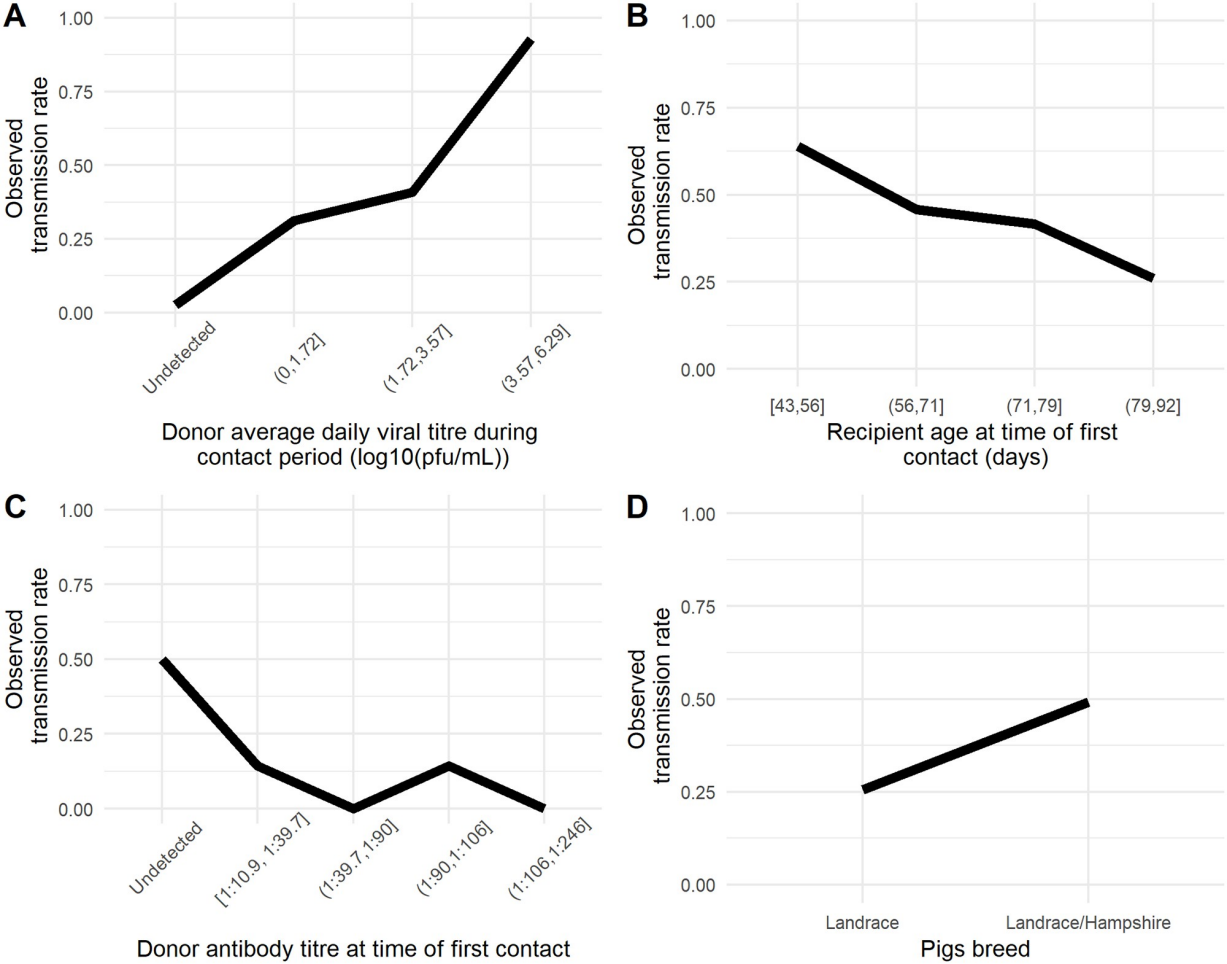
Probability of transmission according to each estimated effect. A. standardized shed virus titre, B. standardized antibody titre, C. Standardized R pigs’ age at time of first contact, D. Pigs’ breed. Donors antibody titre at time of first contact binned as undetected and quartiles for detectable levels; D. Pig breed. The dots represents empirical observations, the black line represent the expected values of the posterior predictive distribution and the grey area represent the 95% uncertainty interval. The values of non-varying predictors were set as the estimated mean.

We fit a Bayesian regression model to estimate the effect of virus and animal properties on the probability of transmission. The best model fits included the following standardized predictors: the average raw shed virus titre of the D pig during the contact period, the D pig antibody titre at the beginning of the contact period, the D and R pigs’ breed and R pig age at time of first contact (Fig 5). The model converged correctly (S2 Fig & Table 2). We evaluated the model fit with leave-one-out cross-validation using Pareto smoothed importance sampling. All Pareto shape parameters were <0.7 and the effective number of parameters was estimated as 3.1 (se = 0.8), lower than the number of parameters. This indicated that the model fits the observations well.

**Fig 6:**
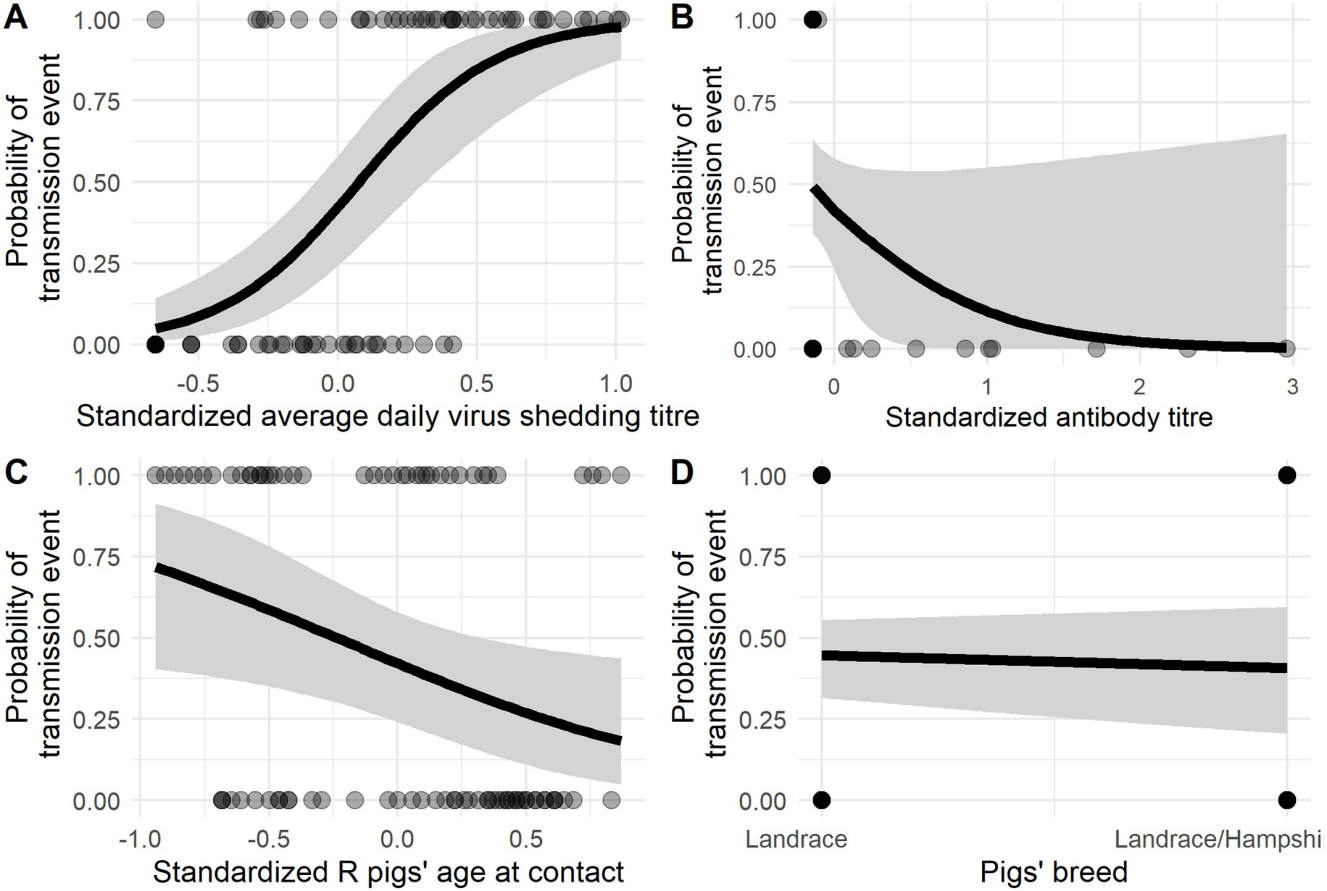
Observed transmission rate according to the putative predictors. A. Donor average viral titre during contact period binned by quartile; B. Recipient age at time of first contact binned by quartile; C. Transmission rates were computed as the ratio of transmission events to all contact times for each variable bin.

Parameters estimates are shown as odds in Table 2. The odds associated with the D pig average shed virus titre during the contact period and with R pig age at time of first contact were 57.0 (95% credible interval—95%CI = [10.6 − 377]) and 0.259, (95%CI = [0.0724 − 0.899]), respectively. The model therefore suggests that younger pigs exposed to higher virus titres have greater odds to be infected (Fig 6). For these two parameters, the 95% credible intervals did not contain the value 1, suggesting a possible true effect of D pig shed virus titre during contact and of age.

D pigs’ level of antibody titres, on the other hand, tended to decrease the odds of transmitting influenza virus (odds = 0.171). However the 95% credible interval contained the value 1 (95%CI = [0.002–1.92]). Similarly for breed, Landrace pigs tended to decrease the odds of transmitting influenza virus (odds = 0.555). However the 95% credible interval contained the value 1 (95%CI = [0.138 − 1.92]) (Table 2 & Fig 6).

### Viral shedding and antibody production modeling

We fit a viral kinetics model to D pigs viral shedding titres. We have tried to fit previously developed mechanistic models [12,16–18], however these models failed to converge. We therefore fit simpler phenomenological models independently to viral titre and antibody titre. In the viral kinetics model, we considered two possible profiles of shedding which we called “peaked” (described by the following parameters: latent period, L_A_, increasing slope, s_1A_, time from infectious contact to the maximal shed virus titre, T_max_ and decreasing slope, s_2A_) and “plateau” (described by the following parameters: latent period, L_B_, increasing slope, s_1B_, time from infectious contact to the begining of the plateau, T_1_, decreasing slope, s_2A_ and time from infectious contact to the end of the plateau, T_2_). We did not make any assumption of the individual viral kinetics profiles. The proportion of pigs showing a peaked pattern, *p*, was unknown and the group to which each pig belongs was defined as the group of highest conditional probability.

The best viral kinetics model included an additive error model and random effects on the following parameters: L_A_, L_B_, T_max_, T_1_, and T_2_. S2 Table shows the maximum likelihood estimates of the parameters. The model fitted well the observed shed virus titres (S2 Fig). The proportion of pigs with a peaked viral kinetics profile was p = 0.475. After a latent period lasting 2.48 days for the peaked and 1.16 days for the plateau profile, shed virus titre increased with slope s_1A_ = 2.17 log_10_(pfu/mL)/day or s_1B_ = 1.16 log_10_(pfu/mL)/day until T_max_ = 4.25 dpc or T_1_ = 2.70 dpc, for peaked and plateau, respectively. Shed virus titre decreased with slope s_2A_ = 0.798 log_10_(pfu/mL)/day after T_max_ for peaked responses or with slope s_2B_ = 1.48 log_10_(pfu/mL)/day after a plateau lasting 2.78 days until T_2_ = 5.48 dpc (S2 Table). The estimated standard deviation of the random effects are also presented in S2 Table.

The estimated onset of antibody production A_1_ was 5.86 dpc (S2 Table).

### Natural history of experimental swine influenza virus infection

The natural history of a disease is described as the course of a pathology from onset to resolution [19] and therefore describes the progression of a disease process in an individual over time, in the absence of treatment.

The percentage of exposed animals that become infected and the latent period varied between the different categories (Table 1). The percentage of exposed contacts that became infected was 100% (21/21) for the S-D contacts and 47% (41/88) for the D-R contacts. The latent period lasted <1 day for the S pigs. For D and R pigs that shed virus the mean latent period was 2.54 (range: 1.00–6.25) days and 3.22 (range 1.00–6.00) days respectively. It should be noted that a latent period >6 dpc would not be detected as recipient pigs were euthanized at 6 dpc.

The other natural history parameters (time to last detected viral titre, duration of shedding, V_max_, T_max_, onset of transmission, generation time Tg (which is an epidemiological parameter representing the mean interval between infection of a primary case and its secondary cases and onset of antibody increase) were only observed in D pigs. An average maximal shed virus titre Vmax = 4.58 ± 0.29 log_10_ pfu/ml was observed at Tmax = 4.51 ± 0.32 dpc in D pigs. Virus was detected for the last time at 7.05 ± 0.28 dpc on average leading to an average duration of shedding of 4.51 ± 0.31 days. The resulting area under the curve (AUC) was estimated as 17.1 ± 1.5 log_10_ pfu/ml. However it is noteworthy that the duration of shedding was right-censored since several pigs did not reach undetectable virus titre at the end of follow up (Fig 3—5D1). The average generation time computed in D pigs was 4.62 ± 0.26 days (range: 2.73–7.42) when computed for shed virus titre in arithmetic scale. Antibody titres were monitored in 19 out of the 21 D pigs (serum samples were not available for pigs 1D2 and 4D2). The latent period and V_max_ in contact pigs (i.e. D pig in S-D contact and R pig in D-R contact) were significantly associated with the daily average shed virus titre. Pigs exposed to higher shed virus titres started to shed virus earlier and shed on average higher shed virus titres (Fig 6). The correlation between the daily average shed virus titre in infectious pig and the latent period in contact pigs was τ = -0.66 (Kendall’s correlation test P<0.0001) and the correlation between the daily average shed virus titre and maximum shed virus titre, Vmax in the contact pig was τ = 0.58 (Kendall’s correlation test P<0.0001).

Antibody was first detected on average at 7.8 ± 0.5 dpc. At the end of the follow-up (9 dpc), the average antibody titre was 1:120.5 ± 1:82.8.

### Comparison of predicted and observed natural history parameters

To assess the accuracy of the model predictions, we simulated viral and antibody kinetics over 100 replicates of the original dataset and computed the relative bias between the natural history parameters computed from the original dataset and the simulated datasets.

**Fig 7:**
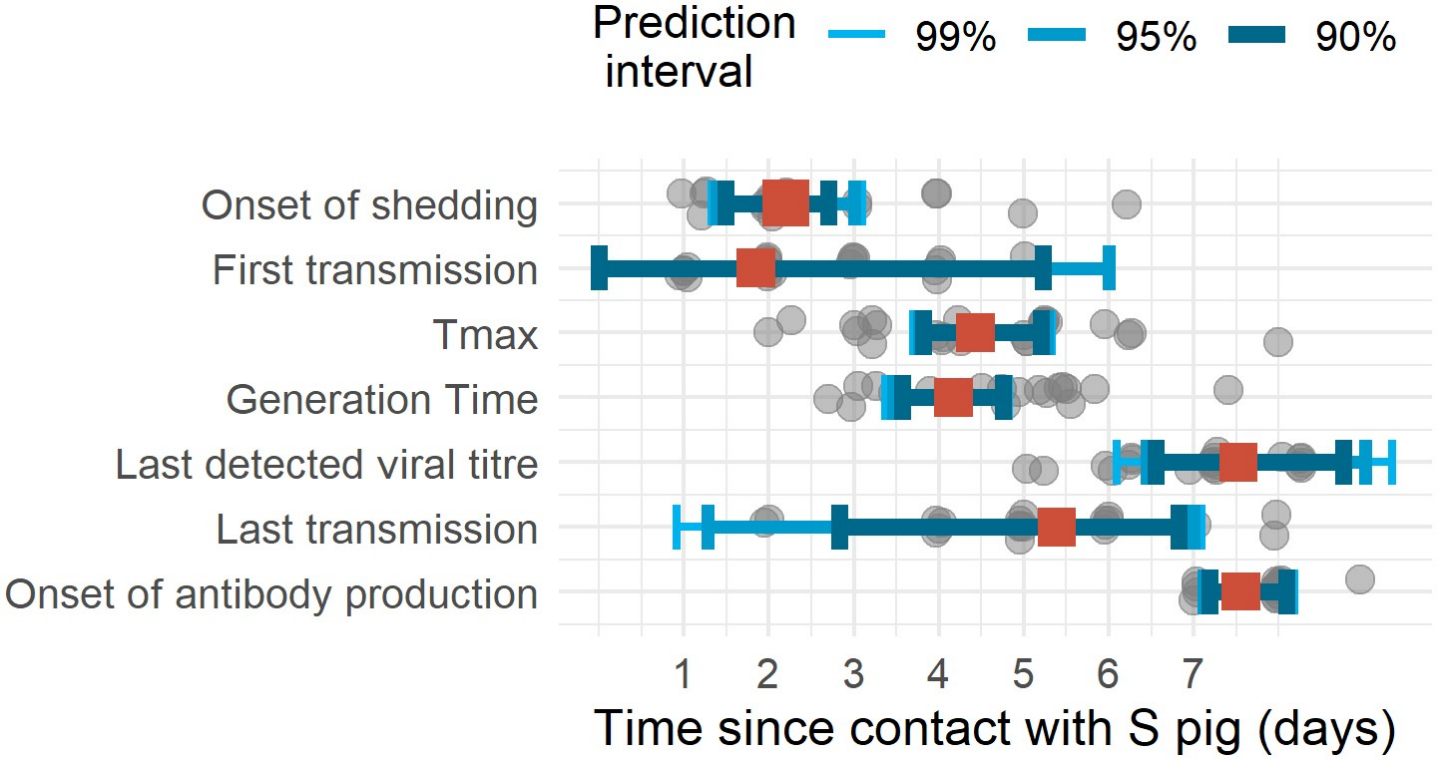
Natural history parameters. Grey dots represent the observed values, red square represent the average simulated replicates mean and the blue intervals, 90%, 95% and 99% prediction intervals computed from 100 replicates of 11 pigs experiments.

For all natural history parameters, the relative bias varied between -20% and +20% (Table 1 & Fig 7). We predict than only 85.8% of pigs would shed virus above the limit of detection. For the latent period, time to last detected virus titre, duration of shedding, time to maximal virus titre, generation time, time to first transmission, transmission period and onset of antibody production, the predicted values were less than 0.5 day different from the observed value, which is shorter than the duration of contact (18 hr).

## Discussion

Experimental transmission studies can provide valuable parameter estimates to inform models of disease spread in animal populations. However, the reliability of these transmission studies depends on the animals transmitting virus receiving a challenge consistent with natural exposure in the field. We established a one-to-one transmission model to accurately estimate transmission parameters of H1N1pdm09 virus in pigs. Donor animals were infected by contact with a seeder pig to mimic naturally acquired infection, with the time of infectious contact known. These donor animals were closely monitored with nasal swabs taken twice daily (before and after contact with the recipient) for shed virus titre measurement and blood samples taken daily for antibody titre measurement. Donor animals were in contact for an 18h period with one recipient pig to allow identification of transmission events and avoid an extended exposure window for the recipient animal.

We found that seeder pigs were shedding virus at very high titers shortly (<24h) after inoculation, while donor pigs started to shed virus between 1 and 6 dpi/dpc which is consistent with experiments where contact pigs were co-housed with animals inoculated with A/swine/Cotes d’Armor/0388/2009 H1avN1 (clade 1C) strain which started to shed virus between 3 and 7 days [20]. This is also consistent with estimates from outbreaks of A/swine/Cotes d’Armor/0388/2009 (H1avN1), A/Swine/Scotland/410440/94 (H1huN2) and A/Swine/Flandres/1/98 (H3N2) in farms where latent period was estimated as 1.4 to 5 days [20]. Noticeably, the latent period of recipient pigs was more than 16 hr longer than in donor pigs. However, the latent period from D pigs was about 2-fold that observed in human volunteer experimental challenge [12,21].

Consequently, the observed time to viral titre peak was also observed later in D pigs than in human experimental infections where it consistently averaged at 2 days post inoculation [12,16,21–23] and the generation time was also longer in D pigs than in human experimental infections where it was estimated as 2.1 or 2.3 days [12,21].

The duration of shedding (4.51±0.31 days) was similar with experiments where 10 unvaccinated contact pigs were co-housed with one pig inoculated with A/swine/Cotes d’Armor/0388/2009 H1avN1 shed virus during 4.50±1.07 days [24] but was shorter than previously reported in another study (6.1 days) [25], however in this latter experiment pigs were co-housed during the whole experiment with two seeder pigs and therefore exposed to high level of shed virus titre for a long period. Interestingly, the duration of shedding was also similar to that reported from a review of volunteer challenge studies were A/H1N1 infection lasted on average 4.50 days [21].

Overall, compared with seasonal A/H1N1 virus experimental infection studies in humans, during which inoculation was most frequently intranasal with the strain A/Texas/36/91 [12,16,21–23], the D pigs started to shed virus later but the duration of shedding was similar.

All these results, suggested that high dose exposure would reduce the influenza latent period. We could not compare the dynamics from human experimental challenge with the S pigs’ influenza dynamics that also received intranasal inoculum because of their short follow-up (2 days).

Virus shedding was, as expected, by far the most important predictor of transmission events. However, despite using an experimental set-up designed to maximize the chances of transmission over an 18 hour period, we only observed transmission on 60% of occasions when virus was detected in the D pig. We note that, importantly, estimates of the transmission period are slightly shorter than the duration of shedding (Table 1). This difference appears to be largely accounted by the lack of transmission (given that virus was detected): on 9/10 and 17/56 occasions when neutralizing antibodies were detected and not detected respectively. There was a single instance of transmission in the presence of antibody. However, the estimated effect of antibody titre with Bayesian logistic regression was not significant. From these results, the role of antibodies remains unclear in the case of primary infection, whether they are blocking the exit of the virus or are only a proxy of late stage infection during which the pigs are less infectious.

We fit a mixture of segmented linear models with random effects to describe the donor pigs viral kinetics and variability. We considered two viral shedding profiles: peaked (47.5% of donor pigs) and plateau (52.5% of donor pigs). Pigs shedding virus according to the plateau pattern shed more, earlier and for a longer period.

We tried and did not succeed to fit mechanistic models as previously used in human, mice and ponies [12,16–18], using mixed effects. This is certainly due to the relatively small number of donor pigs and the high between-subject variability, especially concerning the initial viral titre. In the previous models, the data fitted all came from experimental infection with intranasal inoculation of high doses of virus. Particularly, the latent period in [12] was less variable than observed in the present work. This highlights the importance of developing more realistic transmission experiments.

Recipient pigs exposed to high shed virus titres from donor pigs started to shed virus earlier and at a higher level and therefore had a higher probability of transmitting infection. We also found that younger pigs exposed to higher virus titre during the contact period were at higher risk of being infected. We estimated influenza generation time for the first time in pigs at 4.6 days (with low variance). We note that estimated generation time varies slightly with the scale used to quantify virus titre.

Epidemic models rely on infection kinetics parameters [26]. We therefore combined the viral kinetics model and the Bayesian logistic regression results to predict transmission events in 100 replicated datasets of 11 donor pig populations and to compute natural history parameters. All natural history parameters were accurately predicted with a limited relative bias. Hence, this approach could be used to model viral kinetics and the risk of transmission to predict outbreaks in pig farms and efficacy of control measures such as quarantine of imported pigs. For instance, as we predicted that the time to last detected virus titre is on average 7.52 days post infection, and is shorter than 8.76 days for 95% of the pigs, several scenarios of quarantine for imported pigs could be tested. The effect of the duration of the quarantine (for instance 7, 10 or 14 days) on the protection of the rest of the farm population could be tested. More elaborate quarantine scenarios could also be investigated where the quarantine duration could be adapted considering the detection and possibly the quantification of viral titre, with a cost-effectiveness analyses.

One limitation of our study was that we did not control for the behavior of the donor and contact pigs, though this may have influenced the likelihood of transmission. Our study probably underestimates variability under natural conditions where animals of different ages, socialisation and immune status are co-housed. Also, we cannot determine whether direct contact, aerosols or contact with contaminated surfaces was the most important mode of transmission in our experiments [25,27].

There is a critical need to develop better models to understand influenza A dynamics that more closely mimic events in humans. Pigs and humans are infected by the same subtypes of influenza A viruses and integrally connected in the ecology of influenza A viruses [28]. Pigs have similar virus shedding patterns and pathogenesis when infected with influenza A virus and are an appropriate animal model for studying immunity to and transmission of influenza virus [15,29]. Understanding influenza transmission has application for humans and swine with the mutual benefit of reducing the zoonotic threat and increasing output in pig farming. However, it is noteworthy that in humans, most of the transmission events occur between case and contacts who have experienced prior exposure and sometimes have been vaccinated. We did not explore the impact of pre-existing immunity in the present work.

To our knowledge, this is the first experimental design of one to one transmission using in-contact infected pigs which allowed us to analyze both viral kinetics and transmission events from animals with naturally acquired influenza virus infection. This allowed us to estimate key parameters including the latent period, duration of virus shedding and onset of antibody production and, to relate these to direct observation of precisely timed transmission events. The design generates much more precise data than can be obtained from the observation of natural transmission events, as in studies of influenza virus transmission in humans [30]. In addition, while we expected that the probability of transmission would depend on viral titre and age, we found that the contact pig virus shedding profile depends on the level of exposure to influenza virus. This should be investigated further to quantify the impact that level of exposure and mitigation measures decreasing this level of exposure have on epidemiological parameters such as the generation time, and therefore the timing and severity of influenza epidemics.

## Material and methods

### Ethics statement

Animal experimentation was approved by the Pirbright Institute Ethical Review Board under the authority of a Home Office project licence (70/7505) in accordance with UK Home Office Guidance on the Operation of the Animals (Scientific Procedures) Act 1986 and associated guidelines.

### Transmission experiments

Twelve transmission experiments were performed. Female pigs were used between seven and ten weeks of age (except for transmission 4 where the pigs were 11 weeks and transmission 5 where they were of unknown age). All pigs weighed 18 to 40 kg, were either Landrace or Landrace x Hampshire breed and were obtained from a commercial high health status herd. All pigs were acclimatized for a period of 7 days before the start of the transmission experiment and randomly allocated to become seeders, donors or recipients.

Two “seeder” (S) pigs were intranasally inoculated with a mucosal atomization device (MAD) with 1 x10^7^ pfu of MDCK grown A/swine/England/1353/2009 virus (H1N1pmd09), as previously described [13]. Two days post inoculation (dpi), the two S pigs were put in contact with two “donor” (D) pigs for 24 hr to allow natural infection. The S pigs were then removed and culled. After the 24 h exposure, one D pig was chosen randomly, designated as donor 1 (D1), and placed in contact with a different naïve recipient (R) pig each day for 18 hours (between 2pm and 8 am) during an 8 day period (recipients R1 to R8) (Fig 1A). The other D pig was the companion pig to D1 and was designated as D2. This contact took place in a chamber made from perplex with dimensions 2.46 m x 2.46 m and 1.4 m high at 19°C (Fig 1B). During the remaining 6 hours of each day from 8 am to 2 pm, while the contact room was cleaned, the two donor pigs were co-housed in a separate room. The R pigs were removed at 8 am to a separate room and supplied with a companion on welfare grounds (Fig 1). S, D and R pigs were euthanized with an overdose of pentobarbital after 3, 9 and 6 days of follow-up, respectively.

### Viral load determination

Nasal swabs were collected daily for the S and R pigs and twice daily (at 8 am and 2 pm) from D pigs with the exception of transmission 1, where only the 8 am nasal swabs were taken. Two nasal swabs (one per nostril) were placed into 2 ml of virus transport medium comprising tissue culture medium 199 (Sigma-Aldrich) supplemented with 25mM Hepes, 0.035% sodium bicarbonate, 0.5% BSA, penicillin, streptomycin and nystatin, vortexed, centrifuged to remove debris and stored at -80°C for subsequent virus determination.

Infectious shed virus titres in nasal swabs were determined by plaque assay on MDCK cells. Samples were 10-fold serially diluted and 100 μl of each dilution added to confluent MDCK cells in 12 or 24 well tissue culture plates. After 1 hr, the plates were washed and overlayed with 2 ml 1:3 2% agarose: medium. Plates were incubated at 37°C for 48 hrs and plaques visualized using 0.1% crystal violet.

Blood samples were collected daily from the 24 D pigs and were not collected from S or R pigs. The microneutralization assay was carried out as previously described with the following modifications [31]. Pig sera were heat inactivated for 30 min at 56°C. Dilutions of heat-inactivated pig sera in 50 μl starting at 1:10 were added to 50 μl of homologous H1N1pdm09 and incubated for 2 h at 37°C. The following antibodies were used to stain the fixed monolayer, first layer mouse anti-NP monoclonal antibody Clone AA5H IgG2a (BioRad) followed by the secondary polyclonal goat anti-mouse Ig HRP antibody (Dako). Titers were defined as the serum dilution resulting in 50% reduction in NP expression.

### Estimation of transmission probability

We used a Bayesian logistic regression to quantify the effect of each putative predictor on DR transmission events. We included the following putative population predictors: average shed virus titre (computed from the arithmetic scale) of the D pig during the contact period, D pig antibody titre at the beginning of the contact period, D and R pigs’ breed, R pig age at time of first contact.

Inputs were standardized following Gelman’s recommendations [32,33]. The priors were set as Cauchy distributions of scale 2.5 [33]. We fitted the model using 4 MCMC chains of 10,000 iterations with 10% burn-in. To assess model convergence, we visually inspected each parameter trace looking for a “hairy caterpillar” aspect. Effective sample size > 400 were considered as acceptable. We also performed leave-one-out cross-validation using Pareto smoothed importance sampling implemented in the brms package [34,35].

### Antibody and viral kinetics modeling

To model antibody kinetics, we used a segmented regression with mixed effects described by 2 parameters, A_1_ and A_2_ representing the time to antibody production and the slope of increasing antibody titre, respectively.

Influenza shed virus titre (in pfu/ml) and antibody titre were log_10_- and log_2_-transformed for subsequent analysis, respectively. To model viral kinetics, we used a mixture of segmented regressions with mixed effects. We call *z*_*i*_ the label of the structural model for pig *i*, with M1 for “peaked” (M1) and M2 for “plateau”. In the peaked model, after a latent period lasting L_A_, shed virus titres increase from below the limit of detection to the maximum shed virus titre V_max_ occurring at time T_max_ according to the slope s_1A_ and then decreases according to a slope s_2A_. In the plateau model, after a latent period lasting L_B_, shed virus titre increases from below the limit of detection to the maximum shed virus titre V_max_ according to the slope s_1B_. Shed virus titre remains at V_max_ during the plateau between T_1_ and T_2_, and then decreases according to a slope s_2B_.

We estimated the vector of fixed effects (*μ*), the inter-individual variance-covariance matrix (Ω), the variances (*σ*_*V*_ and *σ*_*A*_, respectively) of the additive error terms for viral kinetics (VK) and antibody kinetics (AK) models and the vector *π* = (*π*_*M*1_, *π*_*M*2_) of probabilities for each virus shedding profile (M1 or M2 here). The random effects and error terms were assumed to follow $N(0,\omega ),\;N(0,\sigma_{V})$ and $N(0,\sigma_{A})$, respectively. We call *θ* = (*μ*, Ω, *σ*_*V*_, *σ*_*A*_, *π*) the vector of population parameters.

The individual parameters were estimated as empirical Bayes estimates. More specifically, the group to which each individual pig belongs was estimated as the group of highest conditional probability: $\hat{z_{i}}=arg\underset{m}{\text{max}}\;P(z_{i}=m|,y_{i},\theta )$, where $\hat{z_{i}}$ is the estimated structural model of pig *i*, *m* = {*M*_1_, *M*_2_} is the label of the structural model, *y*_*ij*_ is the observed shed virus titre of pig *i* and *θ* is the vector of population parameters [36,37].

We censored shed virus titres at the detection limit of 1 pfu/mL and antibody titres at the detection limit of 1:10. For model parameters with a random effect, we assumed that parameters for individual pigs were lognormally distributed about a population log-mean value, with an estimated standard deviation describing the random effect.

Viral kinetics model parameters were estimated using the SAEM (Stochastic Approximation of Expectation Maximization) algorithm implemented in Monolix2018R1 [38]. In a first step, we estimated the parameters with the simulated annealing version of the SAEM algorithm to find the global maximum of the likelihood. During the second step, we re-estimated the parameters using the estimates from step 1 as initial values. We performed model selection using an Akaike Information Criterion (AIC) to determine which kinetics parameters should have random effects, using the rule “the smaller the better”.

### Experimental swine influenza virus infection natural history parameters

We considered that pigs were infected if virus was detected from the nasal swab. In D pigs this was confirmed by the detection of antibody at the end of the experiment.

The natural history of a disease refers to the time course of a disease in the absence of treatment and more specifically to the latent period/incubation, duration of shedding, maximal shed virus titre (V_max_), time to V_max_, onset of transmission, generation time, proportion of exposed contacts who becomes infected [39,40].

We defined the latent period as the interval between inoculation or contact and first detection of virus. The duration of shedding is the interval between the onset of shedding and the last sample time when virus is detected. We defined the onset of antibody production as the time interval between the infection of a pig and the first time antibody is detected. It is noteworthy that the times to first and last virus detection and to first antibody detection are interval censored, therefore the observed latent period is an upper limit whereas the duration of shedding is a lower limit. For instance, the moment of first detectable virus occurred at an unknown time during the time interval between the last sample when shed virus titre was undetected and the first sample when it was detected. Therefore, considering first detection of virus for the definition of latent period means that we consider the upper limit of this time interval.

We considered that a transmission event occured when virus is detected at least once in one of the recipient (R) pigs. We therefore defined the onset of transmission as the time interval between the infection of an individual and the first time when a transmission event is observed from this individual. Here, the onset of transmission and duration of transmission are upper and lower limit values respectively.

Generation time, *Tg* is the period of time between the onset of virus shedding in a primary case and the onset of infectiousness in a secondary case infected by the primary. It has been assumed that the probability to observe a transmission from a D to an R pig at time *t*, also called infectiousness is proportional to D1 pig’s viral shedding at *t*, *V*(*t*) [21,41]. We call *k*_1_ the proportionality constant. We also assumed random contacts between contact donor (D1) and R pigs (i.e. homogeneous mixing independent of infection time course, with a contact rate *k*_2_). Hence, the probability of observing a transmission event at time *t* is given by: *P*(*E* = 1│*t*) = *k*_1_*k*_2_*V*(*t*).

The total amount of virus shed by a D pig is given by the area under the curve, $AUC=\int_{0}^{+\infty }V(x)dx$. Therefore, we can compute the generation time *Tg* as the expectation of *P*(*E* = 1│*t*) by integrating *P*(*E* = 1│*t*)*t* with respect to *t* and normalized by the total rate of transmissions over time, which is *k*_1_*k*_2_*AUC*. Dropping the constants *k*_1_ and *k*_2_ leads to the following expression:

$$Tg=\int_{0}^{+\infty }\frac{t\times V(t)}{AUC}dt$$

All integrals were computed using the trapezoidal rule as implemented in the caTools package [42]. We presented the estimates as mean ± standard error (se) for quantitative variables.

### Assessing model predictions accuracy

To assess the accuracy of the model predictions, we simulated viral and antibody kinetics over 10 days for 100 datasets of 11 pigs, using the mlxR package. Individual parameters were sampled from the estimated population distribution.

Using the viral kinetic profiles simulated for the 11 pigs in each replicated dataset, we simulated the transmission events for each day according to the Bayesian logistic regression estimates.

We then computed the natural history parameters for the 100 replicates and compared their distributions to the parameters computed directly from the observations. To quantify the accuracy of our predictions, we computed the relative bias as $\frac{E[\hat{\theta }]-\theta }{\theta }$, where $\hat{\theta }$ is the mean of the parameter computed from the simulated data for a given replication, $E[\hat{\theta }]$ is the expectation of the within-simulation mean across the 11 simulated viral kinetics of parameter and *θ* the observed mean parameter computed from the original dataset.

## Supporting information

S1 Table Characteristics of pigs with baseline antibody. (DOCX)

S2 Table Viral kinetics and antibody kinetics population parameter estimates. rse stands for relative standard error. The fixed effect represent the average population parameter value and the random effect represents the between pig variability.
(DOCX)

S1 Fig Bayesian logistic regression parameters. Left panels: posterior distribution. Right panels: convergence traces.
(TIFF)

S2 Fig Viral kinetics model fit. Observations are shown by the grey dots and best fit curves by the black lines. Each box represents a donor pig.
(TIFF)

S1 File Raw data. (ZIP)

S2 File Modeling support (R codes and Monolix models). (ZIP)
