# Supplementary material for: Timelines of infection and transmission dynamics of H1N1pdm09 in swine
Source: PLoS Pathog. 2020 Jul 24;16(7):e1008628. doi: 10.1371/journal.ppat.1008628 (PMC7446876; doi:10.1371/journal.ppat.1008628)
Supplement: S1 Table — (DOCX) [file ppat.1008628.s001.docx]

**S1 Table. Characteristics of pigs with baseline antibody**

| Pig ID | Onset of shedding (dpc) | First transmission event (dpc) | Number of transmission events | Duration of shedding (d) | Age (w) | Baseline antibody titre (50% inhibitory titre) | Antibody titre at 9 dpc (50% inhibitory titre) |
| --- | --- | --- | --- | --- | --- | --- | --- |
| 3D1 | 5.25 | 5 | 1 | 1.75 | 9.5 | 1:142.7 | 1:94.2 |
| 2D2* | 2.25 | - | - | 4.75 | 10 | 1:36.2 | 1:57.0 |
| 6D2* | 3.00 | - | - | 5.00 | 9 | 1:56.1 | 1:38.0 |

***** Pigs 2D2 and 6D2 are companion D pigs
