# Supplementary material for: Timelines of infection and transmission dynamics of H1N1pdm09 in swine
Source: PLoS Pathog. 2020 Jul 24;16(7):e1008628. doi: 10.1371/journal.ppat.1008628 (PMC7446876; doi:10.1371/journal.ppat.1008628)
Supplement: S2 Table — rse stands for relative standard error. The fixed effect represent the average population parameter value and the random effect represents the between pig variability. (DOCX) [file ppat.1008628.s002.docx]

**S2 Table. Viral kinetics and antibody kinetics population parameter estimates.** rse stands for relative standard error. The fixed effect represent the average population parameter value and the random effect represents the between pig variability.

|  | Parameter | Description | Fixed effect (rse %) | Random effect % (rse %) |
| --- | --- | --- | --- | --- |
| Peaked | L_A_ | Latency period | 2.48 (17) | 55 (22) |
|  | S_1A_ | Increasing slope | 2.17 (14) | - |
|  | T_max_ | Time from infectious contact to the maximal virus shedding titre V_max_ | 4.25 (8) | 27 (25) |
|  | S_2A_ | Decreasing slope | 0.798 (7) | - |
| Plateau | L_B_ | Latency period | 1.16 (21) | 67 (25) |
|  | S_1B_ | Increasing slope | 3.17 (-) | - |
|  | T_1_ | Time from infectious contact to the begining of the plateau | 2.70 (10) | 32 (27) |
|  | S_2B_ | Decreasing slope | 1.48 (4) | - |
|  | T_2_ | Time from infectious contact to the end of the plateau | 5.48 (4) | 11 (30) |
|  | p | Probability of peaked profile | 0.475 (23) | - |
|  | add_1_ | Additive error for VK model | 0.672 (4) | - |
| Antibody kinetics | A_1_ | Antibody increase onset | 5.86 (0.7) | - |
|  | A_2_ | Slope of antibody increase | 1.25 (22.2) | 71(26.8) |
|  | Add_2_ | Additive error for AK model | 0.23 (8.3) | - |
