# Supplementary figures and images for: Timelines of infection and transmission dynamics of H1N1pdm09 in swine
Source: PLoS Pathog. 2020 Jul 24;16(7):e1008628. doi: 10.1371/journal.ppat.1008628 (PMC7446876; doi:10.1371/journal.ppat.1008628)

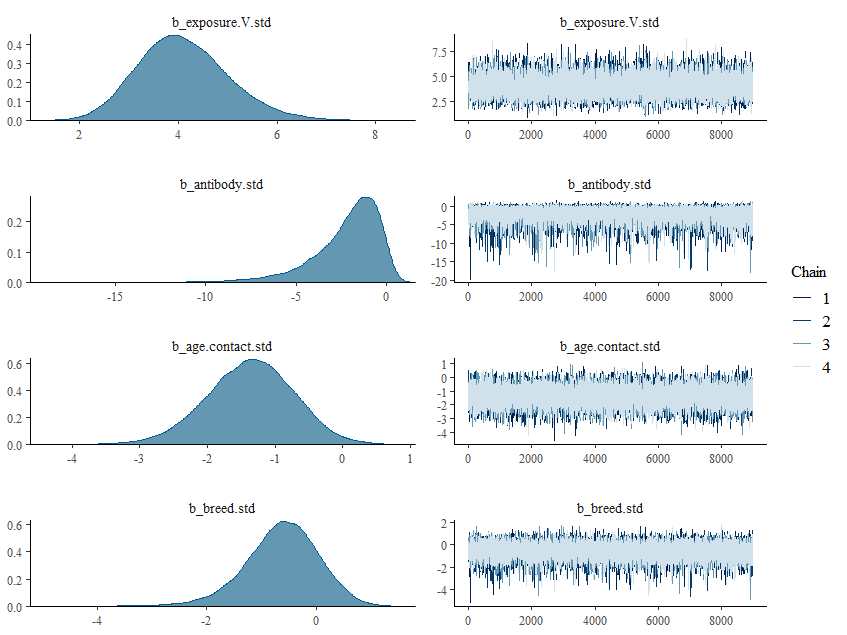

Supplement: S1 Fig — Left panels: posterior distribution. Right panels: convergence traces. (TIFF) [file ppat.1008628.s003.tiff]

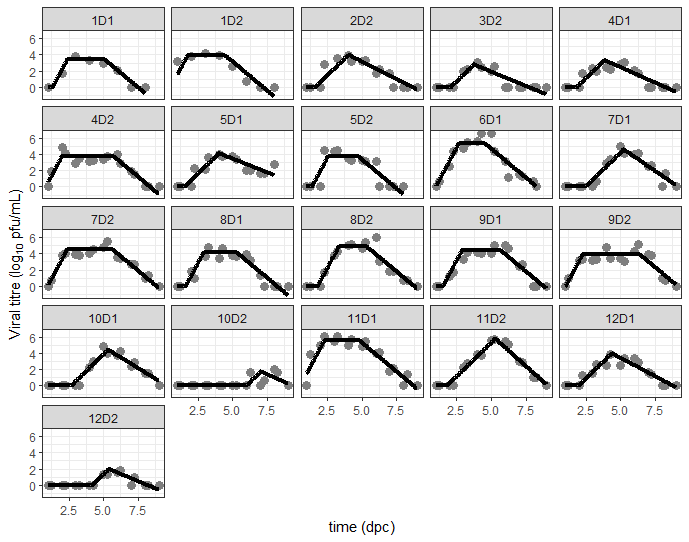

Supplement: S2 Fig — Observations are shown by the grey dots and best fit curves by the black lines. Each box represents a donor pig. (TIFF) [file ppat.1008628.s004.tiff]
